# Proof-of-concept evaluation of next-generation sequencing-based liquid biopsy for non-invasive cancer detection in cats

**DOI:** 10.3389/fvets.2024.1394686

**Published:** 2024-09-13

**Authors:** Carlos A. Ruiz-Perez, Prachi Nakashe, Maggie A. Marshall, Francesco Marass, Tuong Tang, Lisa M. McLennan, Marissa Kroll, Brian K. Flesner, Suzanne Gray, Jill M. Rafalko, Daniel S. Grosu, Susan C. Hicks, John A. Tynan, Dana W.Y. Tsui, Andi Flory, Kristina M. Kruglyak

**Affiliations:** ^1^PetDx, Information Technology, La Jolla, CA, United States; ^2^PetDx, Research Programs, La Jolla, CA, United States; ^3^PetDx, Medical and Clinical Affairs, La Jolla, CA, United States; ^4^PetDx, Analytical Production, La Jolla, CA, United States

**Keywords:** cell-free DNA, genomic alterations, cancer screening, feline lymphoma, multicancer early detection

## Abstract

This proof-of-concept evaluation demonstrates that next-generation sequencing-based liquid biopsy can detect genomic alterations in the blood of cats with cancer and the absence of such alterations in the blood of presumably cancer-free cats. Two cats with cytologically confirmed lymphoma and nine presumably cancer-free cats were included in this analysis. Whole blood was collected from each subject and samples were subjected to DNA extraction, library preparation, and next-generation sequencing. Both cancer-diagnosed subjects had somatic copy number variants (a “cancer signal”) identified in cell-free DNA, suggesting the current presence of cancer in these subjects. All nine presumably cancer-free subjects had unremarkable genomic profiles, suggesting the absence of cancer in these subjects. Liquid biopsy using next-generation sequencing of cell-free DNA allows for blood-based detection of cancer-associated genomic alterations in cats. Such technology has the potential to offer considerable utility in veterinary medicine, particularly for the non-invasive prioritization of small cell intestinal lymphoma versus inflammatory bowel disease in cats with gastrointestinal signs. This study lays the foundation for future studies to fully validate this type of testing for use in clinical practice.

## 1 Introduction

Detecting cancer in cats can be challenging. They are often adept at hiding disease and early manifestations may be subtle, so when obvious clinical signs do emerge, the disease is often advanced (1). Additionally, the clinical presentation of cancer in cats is frequently non-specific (e.g., weight loss, decreased appetite, lethargy) (1), often requiring an extensive evaluation to achieve a diagnosis. These factors, coupled with longer lifespans due to advances in feline medical care and the relative scarcity of clinical trials and novel therapies for the species, have resulted in cancer being the leading cause of death in adult cats (2) with lymphoma, squamous cell carcinoma and soft tissue sarcomas (including injection-site sarcomas) among the most commonly diagnosed malignancies (3).

A non-invasive means of detecting cancer, particularly in cats who appear healthy or have non-specific clinical signs, could offer significant utility in veterinary practice. In humans, blood-based “liquid biopsy” testing has been increasingly utilized for the detection and monitoring of cancer, as well as for genomically targeted treatment selection (4–6). Cancer is fundamentally a “disease of the genome,” and liquid biopsy uses advanced sequencing technology (called next-generation sequencing or “NGS”) to analyze DNA fragments (called cell-free DNA or “cfDNA”) in a blood sample to look for genomic alterations that indicate the current presence of cancer in the patient (7). Such multi-cancer early detection (MCED) tests can detect a variety of cancer types (8, 9).

The feasibility of this type of testing for cancer detection in the veterinary setting was established in a proof-of-concept study in dogs in 2021 (10), and in 2022 a large clinical validation study (11) for a commercially available canine liquid biopsy test (OncoK9; PetDx, La Jolla, CA) demonstrated detection of 30 different cancer types with performance comparable to leading human MCED tests (8, 9). Because genomic alterations are highly specific for cancer, this test was found to have a very low false positive rate and can be employed in a variety of clinical scenarios, including as a screen for dogs at higher risk of cancer, as an aid in diagnosis when cancer is suspected, and for post-diagnosis cancer monitoring (11, 12). Over the past few years, the test has been used clinically for non-invasive cancer detection in thousands of dogs (13), with an in-market study demonstrating real-world performance (14) similar to that established in the validation study.

To expand on previous work in dogs (10–12, 14–16), the present proof-of-concept evaluation was performed to determine whether a novel, NGS-based liquid biopsy test can detect somatic (acquired) genomic alterations (a “cancer signal”) in the blood of cancer-diagnosed cats.

## 2 Methods

Blood samples were collected from 11 cats, including two cancer-diagnosed and nine presumably cancer-free, to evaluate the feasibility of developing an NGS-based liquid biopsy test for cats. Cancer-free cats owned by clinical study site investigators or coordinators, who were deemed to be cancer-free based on lack of history, clinical signs, or exam findings indicating a current or previous suspicion or diagnosis of cancer, were allowed to enroll in the control group; and comorbidities other than cancer, both acute and chronic, were not used to exclude subjects from the control group. PetDx veterinarians reviewed and approved the blood draw procedure for all participating cats. All animal owners provided informed consent prior to sample collection. Neither the blood draw nor test results affected clinical management or outcome for any participating cats.

Each subject had a whole-blood sample collected (7–8.5 mL) using Cell-Free DNA Collection tubes (Roche Diagnostics; Rotkreuz, Switzerland). Subjects were not required to be fasted prior to the blood draw, and no in-clinic processing (e.g., centrifugation, separation, refrigeration, freezing) was required. Samples were shipped to the central testing laboratory (PetDx, La Jolla, CA) at ambient temperature.

Upon receipt at the laboratory, samples were processed with a double-centrifugation protocol to separate plasma from WBCs. Plasma aliquots and WBC pellets were stored at −80°C until they were thawed for testing. Samples were collected between December 2021 and January 2024, and tested between December 2023 and February 2024 using an advanced R&D prototype of an NGS-based liquid biopsy assay for blood-based cancer detection in cats.

In preparation for NGS testing, cfDNA was extracted from plasma using a proprietary bead-based chemistry protocol optimized to maximize cfDNA yield in feline subjects. Genomic DNA (gDNA) was extracted from WBCs using the MagMax DNA Multi-Sample Ultra 2.0 Kit (Thermo Fisher Scientific; Waltham, MA). All DNA libraries were prepared, amplified, and subjected to sequencing for detection of genomic abnormalities using an Illumina NovaSeq 6,000, as previously described in dogs (10, 11).

Sequencing reads were aligned to a domestic cat (*Felis catus*) reference genome (Felis_catus_9.0; GCA_000181335.4) (17). Somatic variant calling was performed using an internally developed bioinformatics pipeline and a proprietary copy number variant (CNV) caller.

## 3 Results

### 3.1 Demographics

The nine presumably cancer-free subjects had no history or clinical signs of cancer at the time of blood draw. They ranged in age from 1.1 to 14 years (median 3.1 years); weights ranged from 3.4 to 7.0 kg (median 5.3 kg); there were 5 males and 4 females; and all were spayed or neutered (Table 1).

**Table 1:** Demographics for the 11 cats evaluated with next-generation sequencing-based liquid biopsy.

Subject ID	Age (years)	Sex	Weight (kg)	Reported breed	Clinical status
PT01	9	MN	5	DLH	Lymphoma
PT02	10	MN	3.6	DMH	Lymphoma
PT03	1.1	MN	3.4	DLH	Cancer-free
PT04	1.7	MN	6.3	DSH	Cancer-free
PT05	1.8	FS	3.4	DSH	Cancer-free
PT06	2.2	FS	5.3	DSH	Cancer-free
PT07	3.1	MN	7.0	DSH	Cancer-free
PT08	3.3	FS	4.1	DLH	Cancer-free
PT09	5.8	MN	5.4	DLH	Cancer-free
PT10	9.3	FS	5.4	DLH	Cancer-free
PT11	14	MN	4.9	DLH	Cancer-free

MN, male neutered; FS, female spayed; DSH, domestic shorthair; DMH, domestic medium hair; DLH, domestic longhair.

Both cancer-diagnosed subjects had a diagnosis of lymphoma. The first subject was a 9-year-old, 5 kg male neutered Domestic Longhair cat with multicentric lymphoma in the peripheral lymph nodes and spleen, diagnosed via lymph node and splenic fine needle aspiration (FNA) cytology. The second subject was a 10-year-old, 3.6 kg male neutered Domestic Medium Hair cat with small cell lymphoma of the gastrointestinal tract, diagnosed via intestinal mass FNA cytology (Table 1).

### 3.2 Results of genomic analysis

**Figure 1:**
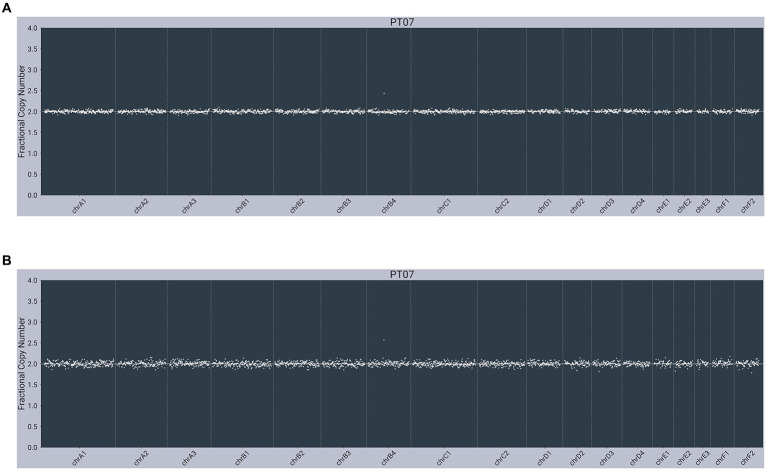
Next-generation sequencing data from a presumably cancer-free, 3-year-old male neutered 7 kg Domestic Shorthair cat (PT07) with unremarkable copy number profiles from: **(A)** plasma cell-free DNA; **(B)** matched genomic DNA from WBC.

All cfDNA and gDNA samples passed quality control metrics, including number of reads and percent alignment. All presumably cancer-free cats were negative for somatic alterations (example provided in Figure 1).

**Figure 2:**
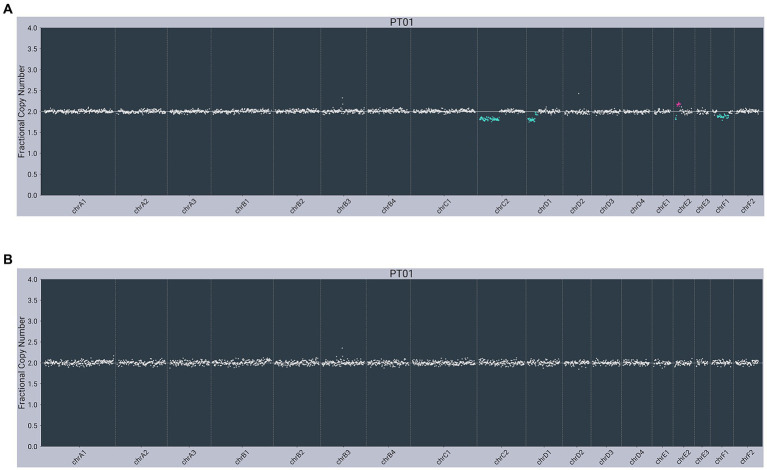
Next-generation sequencing data from a 9-year-old male neutered 5 kg Domestic Longhair cat with multicentric lymphoma (PT01) showing abnormal copy number profiles from: **(A)** plasma cell-free DNA, with partial losses on chrC2, chrD1 and chrF1 and a partial gain/loss pattern on chrE2; **(B)** matched genomic DNA from WBC, with no copy number alterations. *Color added to sequencing traces for visualization of CNVs.*

**Figure 3:**
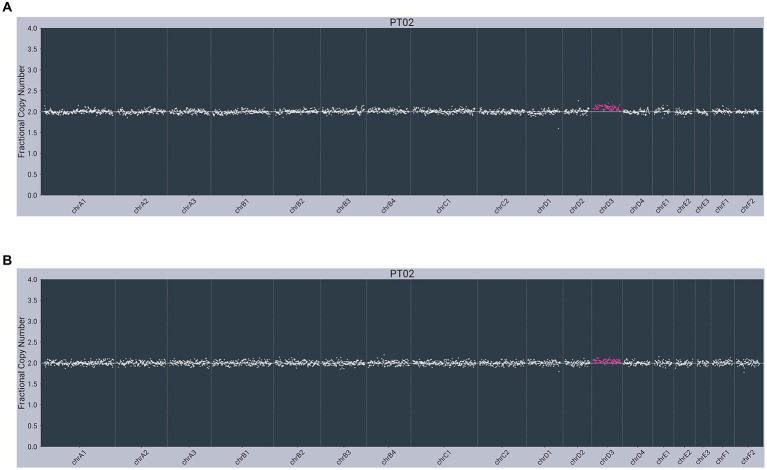
Next-generation sequencing data from an 11-year-old male neutered 3.6 kg Domestic Medium Hair cat with small cell lymphoma of the GI tract (PT02) showing abnormal copy number profiles from: **(A)** plasma cell-free DNA, with a gain on chrD3; **(B)** matched genomic DNA from WBC, with penetration of the same chrD3 gain concomitantly observed in cfDNA. *Color added to sequencing traces for visualization of CNVs.*

In contrast, both lymphoma-diagnosed subjects had somatic alterations (copy number variants, or CNVs) detected in plasma cfDNA. The first subject (PT01) had partial losses observed on chromosome C2 (chrC2), chrD1, and chrF1 and a partial gain/loss pattern on chrE2 in cfDNA, with no abnormalities observed in gDNA (Figure 2). The second subject (PT02) had a gain on chrD3 in cfDNA, with the same gain observed in gDNA (Figure 3).

## 4 Discussion

This proof-of-concept evaluation is the first to demonstrate the feasibility of using NGS-based liquid biopsy to non-invasively detect genomic alterations in plasma cfDNA of cats with cancer. Notably, such alterations were not present in the blood of presumably cancer-free cats in the study. Though larger studies are needed to confirm these observations and to establish assay performance metrics, the ability to detect cancer signal in feline blood using NGS holds the promise of considerable utility in veterinary medicine.

In this study, NGS analysis revealed somatic alterations in cfDNA, which comprises fragmented DNA released through apoptosis or necrosis from healthy cells as well as from cancer cells (if present). The subset of cfDNA originating from malignant tumor cells typically harbors genomic alterations (making it qualitatively different from cfDNA released from healthy cells) and is referred to as “circulating tumor DNA” or “ctDNA.” Genomic DNA isolated from the WBC (buffy coat) layer typically represents the subject’s germline (inherited) DNA and can be used as an intra-subject control when analyzing cfDNA. For the two cancer-diagnosed subjects in this study, genomic alterations were detected in cfDNA, indicating the likely presence of cancer; and in one of these subjects, matching alterations were also found to penetrate into gDNA—a finding occasionally also encountered in lymphoma-diagnosed dogs (11).

The lack of a gold-standard diagnostic work-up to ensure that all presumably cancer-free cats were indeed cancer free is a limitation of this study. Also, the small number of subjects enrolled, while typical for a proof-of-concept study, does not allow for a reliable determination of the likely performance of this testing approach; future studies, enrolling larger numbers of cancer-free cats as well as cats with confirmed cancer diagnoses, will be necessary for NGS-based liquid biopsy to become a clinically validated test for non-invasive cancer detection in feline patients.

The ability to non-invasively detect cancer in pets has many benefits and clinical applications. As liquid biopsy requires only a simple blood draw, there is minimal risk to the patient, and the collection procedure can be performed in most clinical settings. NGS-based liquid biopsy has been shown to detect over 30 cancer types in dogs (11, 14, 18). Although only lymphoma-diagnosed cats were evaluated in the present study, similar multi-cancer detection capabilities should be achievable in cats using an NGS-based approach; this is an area of ongoing research. Additionally, because genomic alterations do not typically occur in non-malignant clinical conditions, NGS-based testing has been employed as an aid in diagnosis for cancer in dogs with various co-morbidities (11, 14); additional research is needed to validate this use case in cats.

Liquid biopsy is currently leveraged across the spectrum of cancer care in dogs: as a cancer screening tool (to detect cancer in high-risk patients with no clinical signs), as an aid in diagnosis (for patients in which cancer is suspected), and as a cancer monitoring tool (for patients previously treated for cancer). The same use cases are anticipated for cats. Perhaps the most anticipated clinical application of NGS-based liquid biopsy in cats is to non-invasively prioritize small cell intestinal lymphoma versus inflammatory bowel disease in patients with gastrointestinal signs. Both conditions are relatively common in cats and have very similar clinical presentations (19, 20), yet to date no single diagnostic criterion or known biomarker reliably differentiates inflammatory lesions from neoplastic lymphoproliferations in the intestinal tract (21).

Studies involving other species (i.e., humans and dogs), have identified limitations of liquid biopsy that are also expected to apply to feline testing. Although not observed in this small proof-of-concept study, it is reasonable to expect that a low rate of false positive results will occur in NGS-based liquid biopsy in cats, as previously observed in large human and canine studies; false negatives can also be expected to occur, as similarly observed in other species (8, 9, 11). False positive and false negative results for this type of testing may result from a variety of biological and analytical factors, including non-malignant proliferation of certain cell lines in older age (such as clonal hematopoiesis of indeterminate potential, or CHIP), constitutional mosaicism, genomic alterations present in one or more fetuses of a pregnant female or in a transplanted organ (including bone marrow), sample swap (at the clinic or laboratory), sample contamination, laboratory processing artifacts, and bioinformatics limitations, among others (7, 11, 14). Detection rates are known to vary by cancer type and stage, and not all cancers are detectable by liquid biopsy (8, 9, 11). As with any single clinical test, liquid biopsy results should not be used as the sole basis for making important decisions such as treatment or euthanasia. Additionally, this type of testing indicates the presence of a cancer-associated signal at the time of blood draw; it does not currently provide information about the risk for future development of cancer (11, 18).

If NGS-based liquid biopsy can be shown to have equivalent performance in cats as in dogs, it has the potential to revolutionize the detection and monitoring of feline cancer, with the ultimate aim of improving clinical management and outcomes. One of the most effective ways to improve outcomes in cancer patients is to detect cancer earlier (i.e., at an earlier disease stage and/or earlier clinical stage) (1, 15, 22–25). Liquid biopsy is one of the most promising tools to date that may help veterinarians achieve this goal.

## Data Availability

The datasets presented in this article are not readily available because they contain proprietary information. Requests to access the datasets should be directed to flory.andi@gmail.com.
